# Mechanical Chest Compressions and Intra-Aortic Balloon Pump Combination for Refractory Ventricular Fibrillation During Primary PCI

**DOI:** 10.1016/j.jaccas.2022.01.018

**Published:** 2022-03-16

**Authors:** Anthony J. Buckley, Cormac O’Connor, Sean Fitzgerald, Terence Hennessy, Thomas Kiernan

**Affiliations:** a Department of Cardiology, University Hospital Limerick, Limerick, Ireland; b Department of Cardiology, Mater Misericordiae University Hospital, Dublin, Ireland

**Keywords:** cardiac arrest, percutaneous coronary intervention, STEMI, mechanical CPR, ventricular fibrillation, CPR, cardiopulmonary resuscitation, CTO, chronic total occlusion, DES, drug-eluting stent, ECG, electrocardiogram, ECMO, extracorporeal membrane oxygenation, LAD, left anterior descending coronary artery, LCx, left circumflex, PCI, percutaneous coronary intervention, RCA, right coronary artery, STEMI, ST-segment elevation myocardial infarction, VF, ventricular fibrillation

## Abstract

This case highlights the successful resuscitation of a 43-year-old man with ST-segment elevation myocardial infarction and refractory ventricular fibrillation by using a combination of mechanical chest compressions and intra-aortic balloon pump insertion. This bailout strategy facilitated primary multivessel percutaneous coronary intervention in a center without on-site extracorporeal membrane oxygenation. (**Level of Difficulty: Advanced.**)

## Central Illustration

**Figure:**
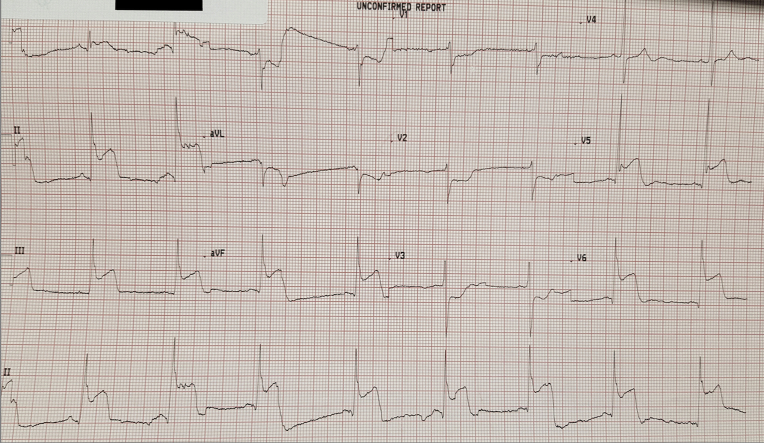

## History of Presentation

A 43-year-old man with a 3-hour history of severe central chest pain drove himself to the emergency department. These events took place in University Hospital Limerick, in the Republic of Ireland, which is a regional primary percutaneous coronary intervention (PCI) center, equivalent to a level II shock center, without any on-site mechanical circulatory support options other than intra-aortic balloon pump (IABP). This hospital is located 200 km from the nearest level I shock center, the only center in Ireland where extracorporeal membrane oxygenation (ECMO) is available.

Learning Objectives

• To appreciate the challenges in managing high-risk STEMIs in centers without ECMO availability.
• To demonstrate a bailout strategy of mechanical chest compressions and IABP insertion for refractory VF during primary PCI.

## Past Medical History

His past medical history was significant only for hyperlipidemia (low-density lipoprotein, 197 mg/dL). He smoked cannabis daily and took no regular medications. Physical examination was notable for the Levine sign and normotension.

## Investigations

**Figure 1:**
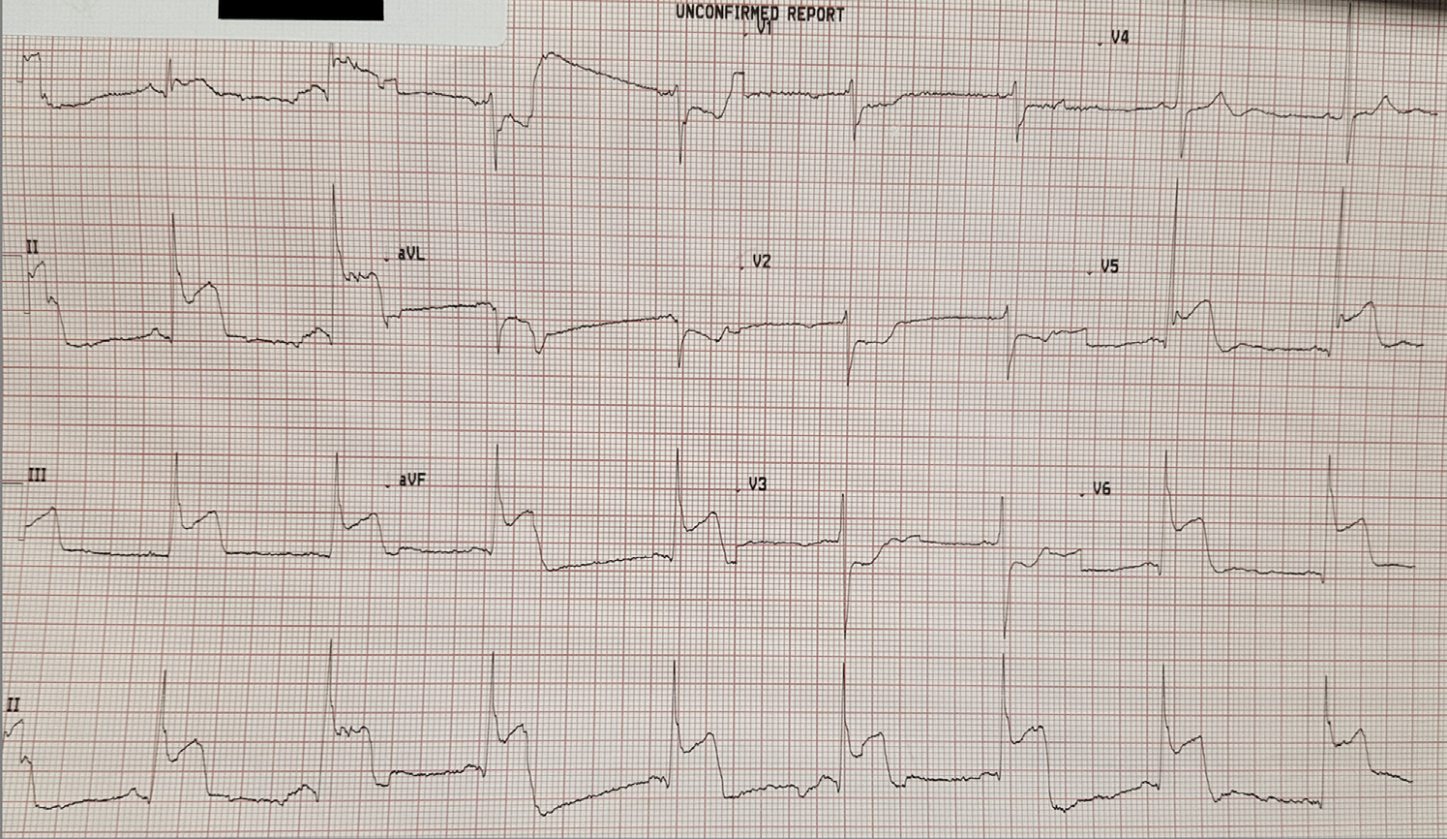
Presenting Electrocardiogram: Inferolateral ST-Segment Elevation Myocardial Infarction With Posterior Extension

The initial electrocardiogram (ECG) revealed an inferolateral ST-segment elevation myocardial infarction (STEMI) with posterior extension (Figure 1). The patient was given a loading dose of aspirin and ticagrelor and was transferred immediately to the cardiac catheterization laboratory.

## Management

**Figure 2:**
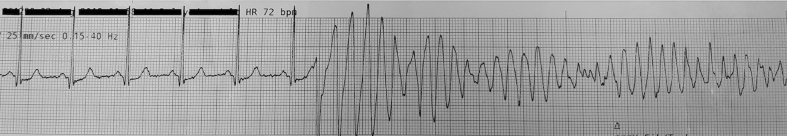
Defibrillator Rhythm Strip Revealing Polymorphic Ventricular Tachycardia

On transfer to the catheterization table, the patient expressed angor animi. The cardiac monitor demonstrated polymorphic ventricular tachycardia (Figure 2), and he was immediately defibrillated with return of spontaneous circulation. This scenario repeated itself several times over the next 2 to 3 minutes, and the patient was treated with further defibrillations, as well as epinephrine and amiodarone boluses, according to the advanced cardiac life support algorithm. The patient persistently reverted to ventricular fibrillation (VF); therefore, a cardiac arrest code was called, and he was intubated by our anesthesia colleagues.

**Figure 3:**
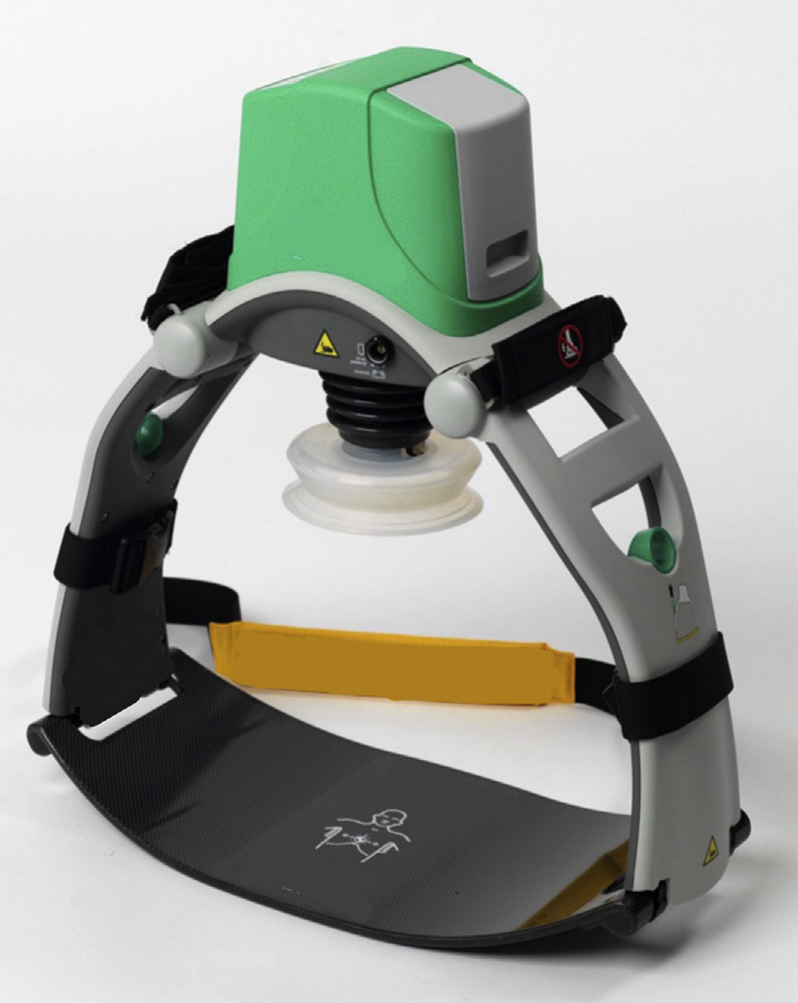
Mechanical Chest Compression System Courtesy of Stryker Medical.

**Figure 4:**
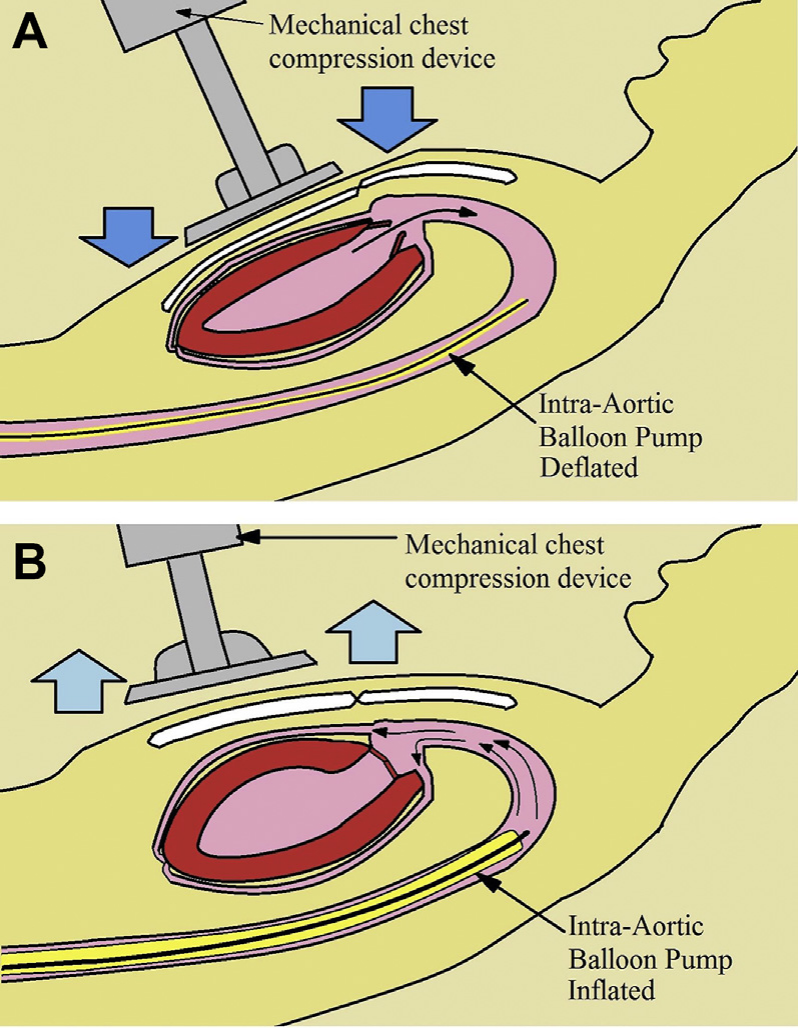
Mechanical Chest Compression System and Intra-Aortic Balloon Pump Use **(A)** The mechanical systolic phase of the cardiac cycle when the chest compression system externally compresses the chest and causes ejection of blood from the left ventricle. The intra-aortic balloon pump is deflated at this stage (after being set to trigger from pressure) and provides some degree of negative pressure in the aorta to facilitate left ventricular emptying. **(B)** The mechanical diastolic phase as the chest compression system is retracted and the chest wall returns to normal position. The heart passively refills with blood from the atria at this stage. Separately, the intra-aortic balloon pump inflates and aims to facilitate coronary perfusion.

During repeated episodes of VF, a mechanical chest compression device (Figure 3) was applied to the patient, and transduction of the right femoral arterial sheath revealed systolic blood pressure of 60 mm Hg during mechanical chest compressions. An IABP was inserted through the left femoral artery, and it was no longer possible to restore sinus rhythm (even transiently). It was not possible to trigger the IABP from the ECG because of the continuous VF, and the IABP was therefore triggered from pressure (Figures 4A and 4B), thus giving an augmented pressure of 80 mm Hg. The decision was made to proceed with angiography with only these circulatory supports in place and the patient in VF throughout the procedure.

**Figure 5:**
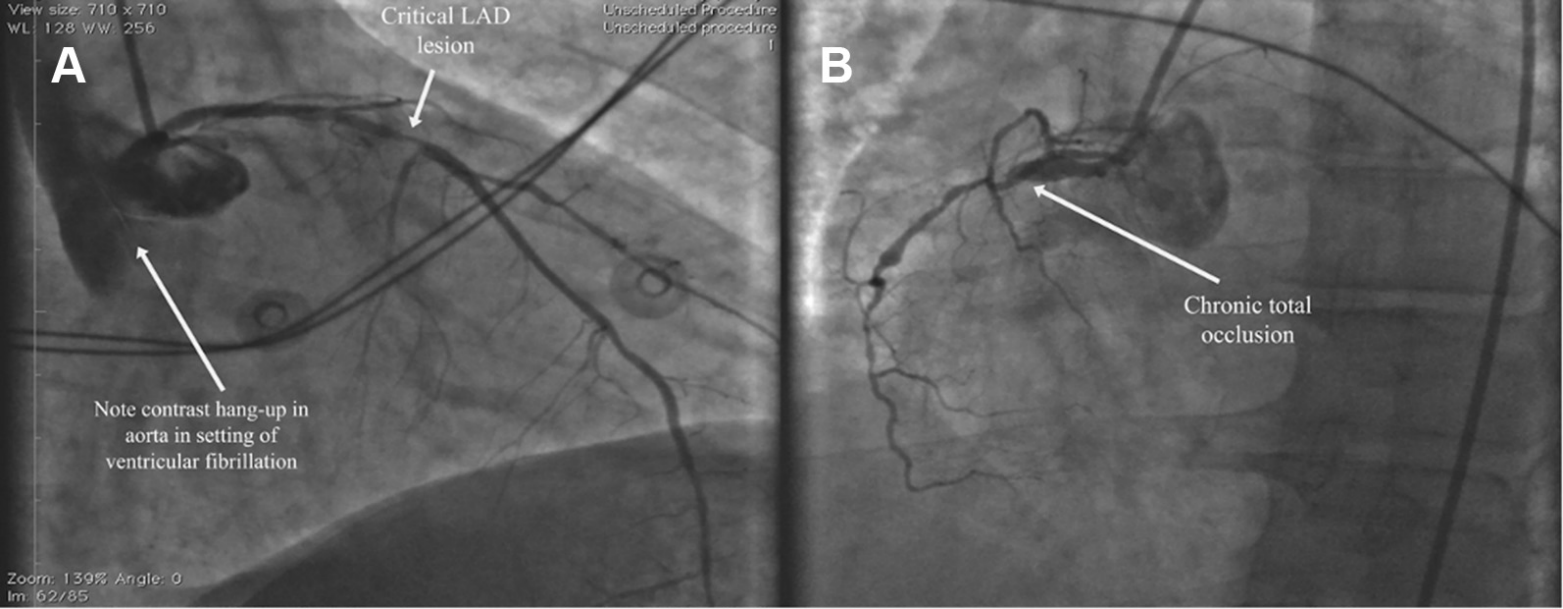
Diagnostic Coronary Angiography During Refractory Ventricular Fibrillation **(A)** Critical mid–left anterior descending (LAD) coronary artery lesion. **(B)** Right coronary artery total chronic occlusion.

Coronary angiography revealed a critical lesion of the mid–left anterior descending (LAD) coronary artery and what appeared to be a proximally occluded left circumflex (LCx) coronary artery and right coronary artery (RCA) chronic total occlusion (CTO) (Figures 5A and 5B, Videos 1 and 2). As evidenced by the angiography films, the patient was in refractory VF. Therapy had been augmented with an amiodarone infusion, and the patient was treated with 17 defibrillation attempts. Our initial approach therefore was to revascularize the LCx artery and restore hemodynamic stability.

**Figure 6:**
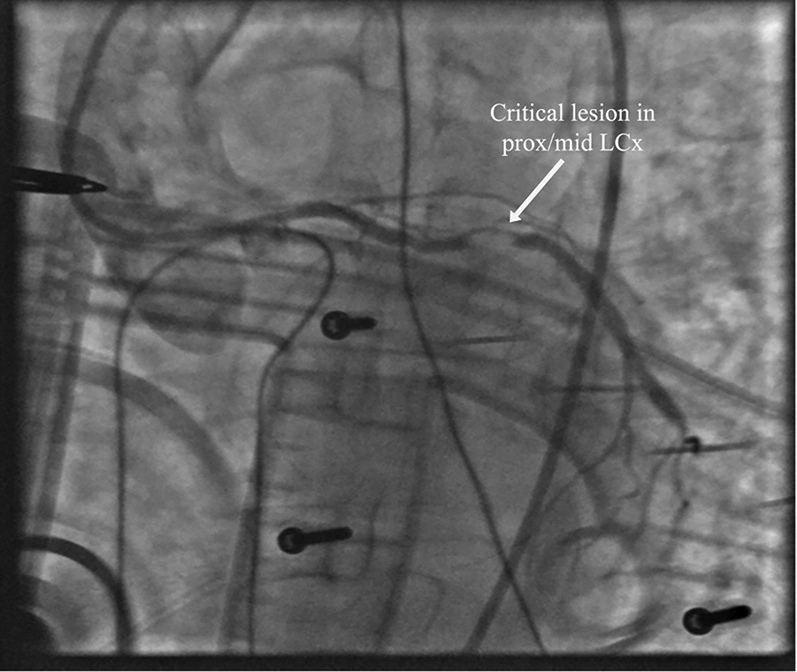
Left Coronary Guide Catheter Revealing Critical Left Circumflex Coronary Artery Lesion LCx = left circumflex; prox/mid = proximal to middle.

**Figure 7:**
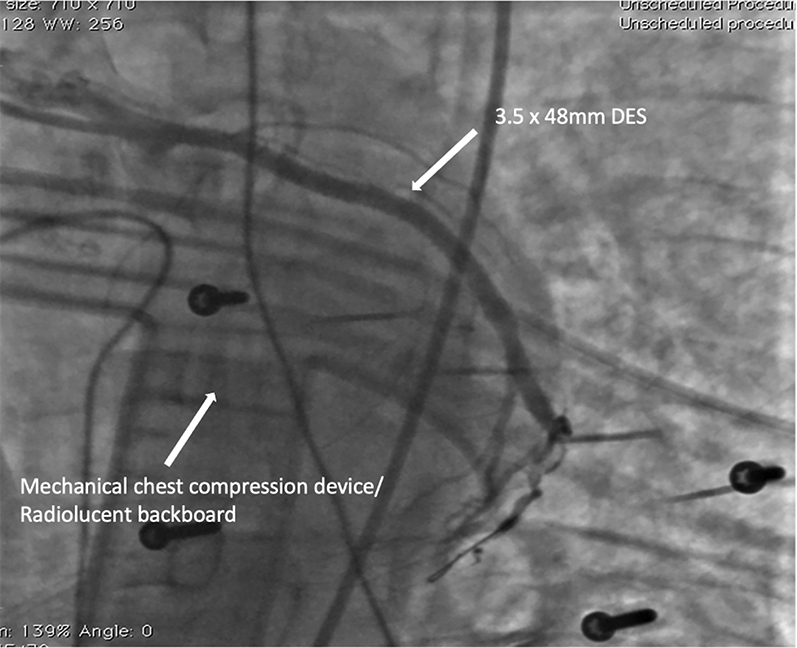
After Left Circumflex Coronary Artery Revascularization DES = drug-eluting stent.

From a right femoral approach, a left coronary guide catheter with an extra backup curve was used to engage the LCx artery. Although initially the LCx artery appeared occluded, angiography with the guide catheter revealed separate LAD and LCx artery ostia, with a critical LCx artery lesion (Figure 6, Video 3). Given the posterior and lateral ECG changes, we elected to treat the LCx artery, which was wired with a 0.014-inch workhorse wire, predilated with a 3.0 × 15 mm semicompliant balloon and stented with a 3.5 × 48 mm drug-eluting stent (DES) (Figure 7, Video 4).

**Figure 8:**
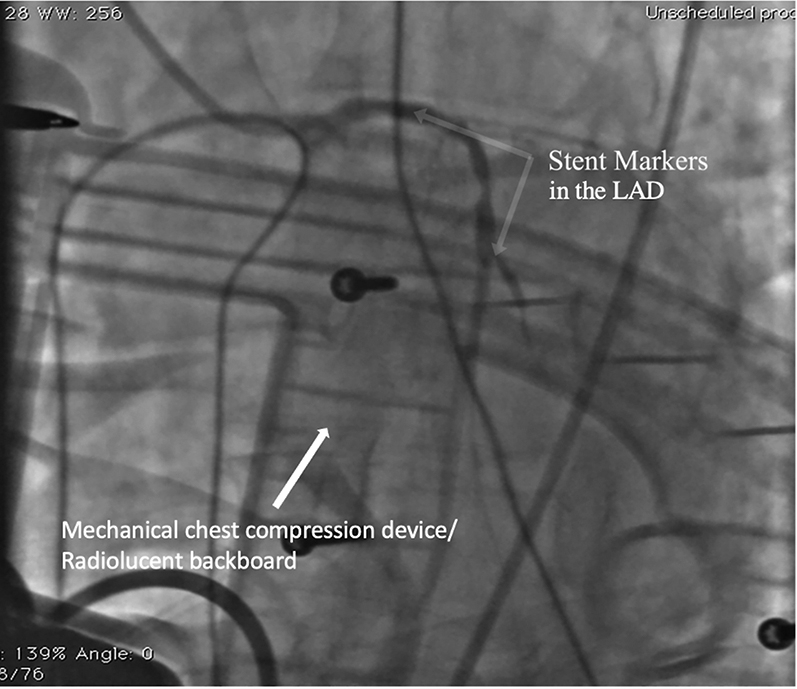
Positioning Left Anterior Descending Coronary Artery Stent During Mechanical Compressions LAD = left anterior descending.

**Figure 9:**
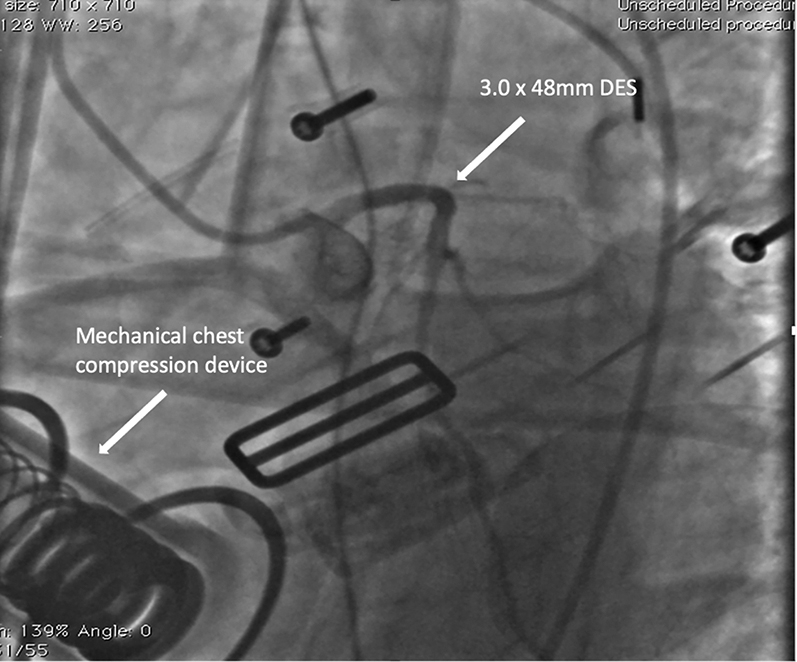
After Left Anterior Descending Coronary Artery Revascularization DES = drug-eluting stent.

Subsequently, the patient was defibrillated from VF to atrial fibrillation. However, his systolic blood pressure remained at 70 to 80 mm Hg despite an additional epinephrine infusion, and the decision was made also to treat the critical LAD lesion during ongoing mechanical chest compressions. A Judkins left 4 guide catheter was used to selectively intubate the LAD artery, which was wired with a 0.014-inch workhorse wire. A 3.0 × 48 mm DES was deployed in the proximal to middle LAD artery, (Figure 8, Video 5), with an excellent angiographic result (Figure 9, Video 6). Following revascularization, the patient was cardioverted to sinus rhythm. At this point, after a 55-minute “code” including 40 minutes with mechanical compressions, we elected to conclude the procedure given that the he had regained a stable rhythm and blood pressure. The patient was then transferred to the intensive care unit and was weaned from vasopressor agents. Removal of the IABP and extubation were performed on postprocedure day 1, and the patient exhibited complete neurologic recovery. The ECG normalized entirely, and the echocardiogram on discharge revealed preserved left ventricular function (Video 7).

## Discussion

Survival of patients with cardiac arrest in the setting of acute myocardial infarction has been shown to improve significantly with venoarterial ECMO, with 1 meta-analysis revealing a 13% absolute increase in survival compared with control subjects (95% CI: 6%-20%; *P* < 0.001; number needed to treat: 7.7).^1^ Unfortunately, these data reflect care at centers with immediate availability of ECMO, and staff trained to facilitate its use, and although impressive, such resources are not available everywhere. This case highlights how a critical situation may be salvaged without immediate advanced mechanical circulatory support.

The CULPRIT-SHOCK (Culprit Lesion Only PCI Versus Multivessel PCI in Cardiogenic Shock) trial supports culprit-only revascularization over multivessel revascularization during the index procedure, both in terms of 30-day and 1-year results principally driven by a difference in mortality,^2^^,^^3^ and it was originally believed that only the LCx artery was occluded in our patient. However, subsequent guide catheter angiography revealed that both the LCx and LAD arteries had severe stenoses, but were in fact open. Thus, we believed that, in the absence of a single definite culprit lesion and in the setting of ongoing hemodynamic instability, it was reasonable to treat both lesions during the index presentation. Notably, the RCA was not revascularized because it appeared diffusely diseased and had a CTO. The inferior ECG changes improved after stenting the LAD and LCx arteries, and we believe that these changes were secondary to occluded left-to-right collateral vessels. Furthermore, the ischemic VF ceased after treating the LAD and LCx arteries, a finding suggesting that they were more likely to be the acute lesions.

The LUCAS 2 Chest Compression System (Stryker Medical) (Figures 3 and 4) is an automated device that provides consistent, high-quality cardiopulmonary resuscitation (CPR), and it facilitated primary PCI in the setting of ongoing chest compressions without exposing those clinicians performing CPR to excessive radiation. Additionally, the radiolucent backboard minimizes the challenges of visualization of the coronary arteries during angiography. The measures that were used to support the patient’s circulatory system were initiated within 2 to 3 minutes (including mechanical compressions, epinephrine bolus, and IABP), with minimal interruption of cerebral perfusion. If the team is extremely familiar with ECMO, it typically takes several minutes to prime the lines, and it takes longer if the team is less familiar with ECMO circuits. Despite the lack of evidence in terms of mortality of mechanical chest compression systems,^4^^,^^5^ combined with the lack of evidence for the use of IABP, we believe that in concert the measures served a particularly pragmatic role in this setting.

## Follow-Up

The patient underwent staged PCI of the RCA with no residual angina at follow-up.

## Conclusions

ECMO remains the gold standard for managing refractory VF during STEMI. An alternative bailout strategy of mechanical chest compressions and IABP insertion may be a pragmatic solution when other advanced circulatory supports are unavailable.

## Funding Support and Author Disclosures

The authors have reported that they have no relationships relevant to the contents of this paper to disclose.

## Appendix

Supplemental Video 1 RAO Cranial View Revealing Critical Mid-LAD Lesion

Supplemental Video 2 LAO View Revealing RCA CTO

Supplemental Video 3 Left Coronary Guide Catheter Revealing Critical LCx Lesion

Supplemental Video 4 After LCx Revascularization

Supplemental Video 5 3.0 × 48 mm DES Deployed in Proximal to Mid-LAD

Supplemental Video 6 After LAD Revascularization

Supplemental Video 7 Transthoracic Echocardiogram Showing Preserved LV Function
